# Bloodstream infections in allogeneic haematopoietic cell recipients from the Swiss Transplant Cohort Study: trends of causative pathogens and resistance rates

**DOI:** 10.1038/s41409-022-01851-y

**Published:** 2022-10-30

**Authors:** Mihaela Sava, Veronika Bättig, Sabine Gerull, Jakob R. Passweg, Nina Khanna, Christian Garzoni, Bernhard Gerber, Nicolas J. Mueller, Urs Schanz, Christoph Berger, Yves Chalandon, Christian van Delden, Dionysios Neofytos, Susanne Stampf, Fabian C. Franzeck, Maja Weisser, Swiss Transplant Cohort Study

**Affiliations:** 1 Division of Infectious Diseases and Hospital Epidemiology, University Hospital Basel, Basel, Switzerland; 2 Division of Oncology, Hematology and Transfusion Medicine, Kantonsspital Aarau, Aarau, Switzerland; 3 Department of Hematology, University Hospital Basel, Basel, Switzerland; 4 Clinic of Internal Medicine and Infectious Diseases, Clinica Luganese Moncucco, Lugano, Switzerland; 5 Clinic of Hematology, Oncology Institute of Southern Switzerland, Ente Ospedaliero Cantonale, Bellinzona, Switzerland; 6 Division of Medical Oncology and Hematology, University Hospital Zurich, Zurich, Switzerland; 7 Division of Infectious Diseases and Hospital Epidemiology, University Hospital Zurich, Zurich, Switzerland; 8 Division of Infectious Diseases and Hospital Epidemiology, University Children’s Hospital Zurich, Zurich, Switzerland; 9 Division of Hematology and Faculty of Medicine, Geneva University Hospitals and University of Geneva, Geneva, Switzerland; 10 Transplant Infectious Diseases Unit, University Hospitals Geneva, Geneva, Switzerland; 11 Clinic for Transplantation Immunology and Nephrology (Swiss Transplant Cohort Study), University Hospital of Basel, Basel, Switzerland

**Keywords:** Haematological cancer, Health care

## To the Editor:

Bloodstream infection (BSI) is the most common infectious complication after allogeneic haematopoietic cell transplantation (HCT) affecting 20–60% of patients in the pre- and early post-engraftment phase [1–3] and later in patients acute graft-versus-host disease (aGvHD) [4]. While quinolone prophylaxis during aplasia is beneficial to some extent, breakthrough BSIs caused by multidrug-resistant (MDR) bacteria have been reported [2, 5]. In this prospective study, we assessed the incidence and risk factors for BSIs in HCT recipients included in the Swiss Transplant Cohort Study (STCS) and the European Society for Blood and Marrow Transplantation (EBMT), the temporal dynamics of causative pathogens and resistance, and risk factors for mortality. The STCS enrolls >95% of all allogeneic HCT recipients in Switzerland [6] and the EBMT enrolls all allogeneic transplant patients in Europe [7]. From a merged dataset of the two cohorts, we included allogeneic HCT recipients aged ≥16 years, transplanted between September 2009 and October 2018 with signed informed consent. The study was approved by the ethics committees of all three transplant centers and the STCS scientific board. As per local guidelines, none of the transplant centers administered quinolone prophylaxis. Primary fluconazole prophylaxis was administered in two centers and all three initiated mold-active antifungal prophylaxis in patients with aGvHD on high-dose steroids. BSIs occurring up to 14 days before the first transplantation were recorded. We defined a BSI as all positive blood cultures with the same pathogen up to 7 days after the first detection. For polymicrobial BSI, all pathogens detected within 5 days from the first positive blood culture were attributed to the same BSI. Skin commensals were considered relevant if isolated from two or more blood cultures within 48 h and the presence of clinical signs and symptoms of infection. Pathogen identification and susceptibility testing were interpreted according to the European Committee on Antimicrobial Susceptibility Testing. We compared two time periods by dividing the follow-up period in half using the date of the median number of transplantations (February 4, 2015). Neutropenia was defined as a neutrophil count <0.5 × 10^9^/L. aGvHD was graded according to standard criteria. The peri-transplantation period was defined as day –14 to day +30 after HCT, the early post-transplantation period day +31 to +100, and the late post-transplantation phase >100 days. Statistical methods are described in the Supplementary materials.

Out of 1688 allogeneic HCT performed, we included 1432 (84.8%) in 1364 patients (Supplementary Fig. S1). At first transplantation, the median patient age was 53 years (IQR 42–61), the most common underlying condition was acute leukaemia (725 patients; 53.2%) and most patients (1298; 95.2%) underwent a single allogeneic transplantation (Supplementary Table S1). During the median follow-up time of 1.88 years (IQR 0.75–4.03), 451 (33.1%) patients suffered from at least one BSI leading to a cumulative BSI incidence of 17.8% (95% CI 15.8–19.9) in the first 30 days and 20.5% (95% CI 18.4–22.7) in the first 100 days (Supplementary Fig. S2a, b). The highest BSI incidence was in the peri-transplantation phase of the second transplant (30.6%; 95% CI 19.9–41.9). This high BSI rate of about one-third of our patients occurring mostly within the first 30 days after HCT has been reported from other centers (12–55%) [8, 9], also those using quinolone prophylaxis [1–3].

**Fig. 1:**
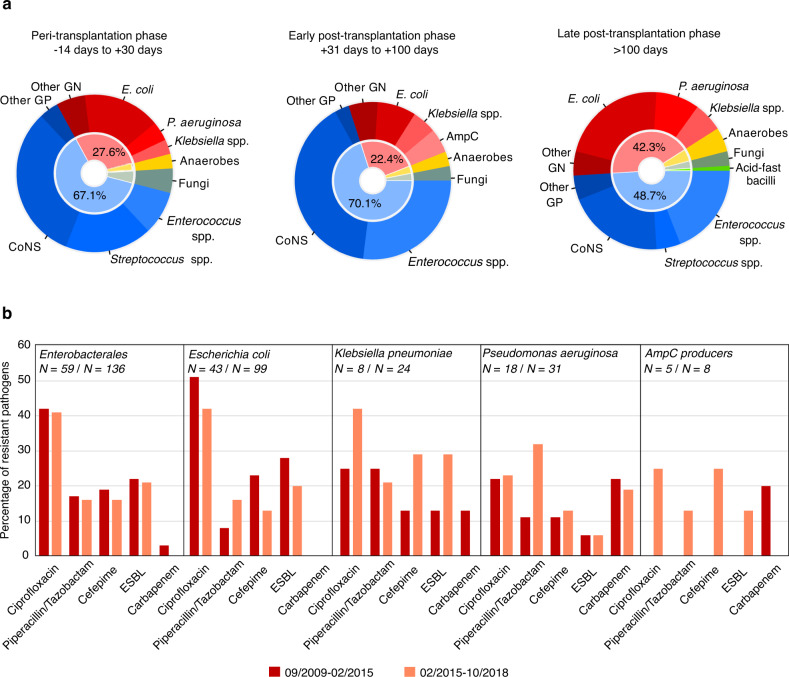
Distribution and resistance patterns of the pathogens causing bloodstream infections in HCT recipients. **a** Pathogens are grouped according to the transplantation timeline. The peri-transplantation phase depicts BSI occurring up to 14 days pre-transplantation through 30 days post-HCT. Early and late post-transplantation phases depict BSI occurring between 31 and 100 days, and beyond 100 days post-transplantation, respectively. Inner circle shows the proportion of GN (in red) and GP (in blue) bacteria isolated. **b** The proportion of resistant isolates among gram-negative bacteria is presented individually for the three most common ones: *E. coli, P. aeruginosa* and *K. pneumoniae*, and grouped for all *Enterobacterales* and AmpC producers. The antimicrobial resistance is depicted in comparison over time by dividing the follow-up period into half using the median number of transplantations, which was achieved on the 4th of February 2015. GN gram-negative bacteria, GP gram-positive bacteria, CoNS coagulase-negative Staphylococci, ESBL extended-spectrum beta-lactamases, AmpC inducible beta-lactamases producing bacteria class C, spp. species.

Patients’ characteristics with and without BSI are described in Supplementary Table S1. Overall, 199 (25.5%) BSIs were polymicrobial. Within 454 gram-positive bacteria (58.1%), coagulase-negative *Staphylococci* (CoNS) were predominant in the peri- and early post-transplantation phases (103; 32.0% and 27; 40.3%, respectively; Fig. 1a), followed by *Enterococcus faecium* isolated mostly in the early post-transplantation phase (18; 26.9%) and *Streptococcus* spp. occurring primarily in the peri-transplantation phase (57, 17.7%). Within 270 gram-negative bacteria (34.6%), *Escherichia coli* was the most common pathogen (142; 18.2%), notably in the late post-transplantation phase (85; 21.7%), followed by *Pseudomonas aeruginosa* (49, 6.3%) and *Klebsiella* spp. (32, 4.1%). Fungi were detected in 28 (3.6%) BSI, distributed evenly across the three phases with a predominance of non-*albicans Candida* spp. (20, 86.9%).

Over time, BSI frequency with *Enterobacterales* increased from 59 (18.3%) to 136 (29.6%) and CoNS decreased from 107 (33.2%) to 103 (22.4%), during the first to the second time period, respectively (Supplementary Table S2). No significant changes in resistance in gram-positive pathogens were noted between the two time periods (Supplementary Table S2). The decrease in gram-positive BSIs over time has also been reported by the ONKOKISS surveillance with a parallel increase in gram-negatives [10], which we could confirm in *Enterobacterales*. No methicillin-resistant *Staphylococcus aureus* and only one vancomycin-resistant *E. faecium* isolate were isolated. These findings support avoiding vancomycin administration as initial empirical therapy for febrile neutropenia in our setting.

Among 195 *Enterobacterales*, 13 (6.7%) isolates exhibited an inducible chromosomal AmpC, and 41 (21.0%) were extended-spectrum beta-lactamase (ESBL)-producing bacteria (1st period 13 isolates; 22.0%; 2nd period 28 isolates, 20.6%; Fig. 1b). Quinolone resistance was observed in 81 (41.5%) isolates and resistance to fourth-generation cephalosporins in 33 (16.9%), both stable over time. No carbapenemase-producing bacteria were detected. Among 49 *P. aeruginosa* isolates, 11 (22.2%) exhibited quinolone- and 10 (20.4%) carbapenem-resistance with an increase in piperacillin/tazobactam resistance between the time periods (1st period 11.1% and 2nd period 32.2%). Four (8.2%) of *P. aeruginosa* isolates exhibited resistance to all three first-line therapy beta-lactam regimens. All *C. albicans* isolates, but five (25%) of non-*albicans Candida* spp. were susceptible to fluconazole.

In uni- and multivariable analysis, factors associated with a first BSI (Supplementary Table S3) were grade III/IV aGvHD, progression/relapse of the hematological disease, preexisting comorbidities, and male gender. Neutrophil recovery was a protective factor for the occurrence of BSI. One-year post-transplant mortality was 26.6% (95% CI 24.3–29.06); 46 (10.2%) died within 21 days from the first BSI. The median survival time was 5.85 years (95% CI 4.2–9.1) (Supplementary Fig. S3) with the main cause of death being progression/relapse of the hematological condition (329; 58.6%, data not shown).

Factors associated with mortality (Supplementary Table S4) were hematological progression/relapse (HR 9.4, 95% CI 7.41–11.91, *p* < 0.001) and occurrence of BSI, modeled using time-varying coefficients: BSI had a HR of 5.95 (95% CI 2.38–14.86, *p* < 0.001) in the peri-transplantation, 4.41 (95% CI 2.33–8.34, *p* < 0.001) in the early post-transplantation, and 10.7 (95% CI 7.79–14.69, *p* < 0.001) in the late post-transplant period.

The strengths of the current study are the representative and complete data, and the analysis of the BSI according to the different HCT time periods—including a long follow-up time beyond neutropenia. Limitations are the observational nature of data, lack of individualized data on previous antibiotic exposure, and colonization with MDR pathogens.

To conclude, BSI occurred in one-third of HCT recipients, most frequently in the first 30 days post-transplantation. The observed quinolone resistance of 41.5% in *Enterobacterales* and 22.4% among *P. aeruginosa* in our study, together with resistance rates of 56 and 53% reported by transplantation centers across Europe [11] and MDR breakthrough infections [5] argue against the benefit of quinolone prophylaxis in similar epidemiological settings.

Considering the low rate of BSI with ESBL-producing or carbapenem-resistant pathogens (3% and <1%, respectively) among our patients, a de-escalating approach, as recommended by the ECIL-4 guidelines [12], using carbapenems for all febrile episodes might not be justified in our setting. Rapid identification of pathogens and susceptibility phenotype remain key to choose an efficient regimen at a minimal cost of selection pressure.

## Supplementary information

Supplementary Content

## Data Availability

Data are available on reasonable request. STCS data are open for researchers and the data access is regulated by the STCS scientific committee.
