# Supplementary material for: Bloodstream infections in allogeneic haematopoietic cell recipients from the Swiss Transplant Cohort Study: trends of causative pathogens and resistance rates
Source: Bone Marrow Transplant. 2022 Oct 30;58(1):115–8. doi: 10.1038/s41409-022-01851-y (PMC9812769; doi:10.1038/s41409-022-01851-y)
Supplement: Supplementary file 1 — Supplementary Content [file 41409_2022_1851_MOESM1_ESM.docx]

**Supplementary Content**

**Statistical analysis**

Continuous variables were summarized using median and interquartile ranges and categorical variables with counts and frequencies. P values for comparisons of descriptive statistics were derived from the Mann–Whitney U test for continuous variables and the chi-square test for categorical variables. For time to event analysis, the starting point was 14 days prior to the date of cell infusion/transplantation.

Kaplan–Meier estimates were used to plot overall survival probabilities. The cumulative incidence function was used to estimate the probability of experiencing a first BSI episode, considering death without BSI as a competing event (1). We applied uni- and multivariable Cox proportional hazard models to assess the association of prespecified variables on the time to outcome events. For the outcome of first BSI in the presence of the competing risk of death, cause-specific hazard ratios from Cox models were estimated. If multiple assessments of covariates during the observation period could be derived from the dataset, they were modelled as time- dependent covariables (number of HCT, aGvHD grade, Karnofsky score at each transplantation, neutrophil engraftment, haematological disease progression/relapse, and BSI). In the case of the predictor variable BSI in the model of mortality, the time-dependent variable had a value of “present” from the date of onset of every BSI until 28 days later. To test the assumption of proportional hazards, we used Schoenfeld residuals. In case the proportional hazards assumption was violated for individual covariates, we introduced step functions to allow for time-varying coefficients for affected variables (i.e., modelling varying effect sizes for those variables depending on the point of time in the follow-up) (2). Results are presented as hazard ratios (HR) and 95% confidence intervals (CIs). The complete case analysis approach was used to handle missing data. All statistical analyses were performed using Stata Version 16.2 (StataCorp, College Station, Texas, USA).

**References:**

1. Fine JP, Gray RJ. A Proportional Hazards Model for the Subdistribution of a Competing Risk. Journal of the American Statistical Association. 1999;94(446):496-509.

2. Zhang Z, Reinikainen J, Adeleke KA, Pieterse ME, Groothuis-Oudshoorn CGM. Time-varying covariates and coefficients in Cox regression models. Ann Transl Med. 2018;6(7):121-.

**Table S1: Characteristics of HCT recipients at first transplantation**

|  | **No BSI**  **N = 913**  **(66.9%)** | **BSI**  **N = 451 (33.06%)** | **Overall**  **N = 1 364**  **(100%)** | **p- value** |
| --- | --- | --- | --- | --- |
| **Patients** | | | | |
| Male gender | 543 (59.5) | 291 (64.5) | 834 (61.1) | 0.072 |
| Age at 1^st^ HCT, years (IQR) | 53 (42 – 61) | 53 (41 – 61) | 53 (42 – 61) | 0.96 |
| Karnofsky Score |  |  |  |  |
| ≥ 90 | 763 (83.6) | 358 (79.4) | 1 121 (82.2) | 0.11 |
| Underlying condition |  |  |  | 0.39 |
| Acute leukaemia | 479 (52.5) | 246 (54.5) | 725 (53.2) |  |
| Chronic leukaemia | 54 (5.9) | 28 (6.2) | 82 (6.0) |  |
| Lymphoma | 101 (11.1) | 45 (10.0) | 146 (10.7) |  |
| MDS/ MPN | 204 (22.3) | 86 (19.1) | 290 (21.3) |  |
| Plasma cell disorders | 54 (5.9) | 28 (6.2) | 82 (6.0) |  |
| Other° | 21 (2.3) | 18 (4.0) | 39 (2.9) |  |
| Number of allogeneic HCT |  |  |  | **<0.001** |
| 1 | 894 (97.9) | 404 (89.6) | 1 298 (95.2) |  |
| 2 | 17 (1.9) | 47 (10.4) | 64 (4.7) |  |
| 3 | 2 (0.2) | 0 | 2 (0.1) |  |
| Interval from 1^st^ to 2^nd^ transplant, days (IQR) | 578 (461 – 785) | 418 (246 – 663) | 517 (258 – 665) | 0.096 |
| Graft source |  |  |  | 0.17 |
| Peripheral blood | 806 (88.3) | 385 (85.4) | 1 191 (87.3) |  |
| Bone marrow | 104 (11.4) | 62 (13.7) | 166 (12.2) |  |
| Cord blood | 3 (0.3) | 4 (0.9) | 7 (0.5) |  |
| Donor: |  |  |  | **0.038** |
| Related | 429 (47.0) | 189 (41.9) | 618 (45.3) |  |
| Unrelated | 478 (52.4) | 262 (58.1) | 740 (54.3) |  |
| Syngeneic | 6 (0.7) | 0 | 6 (0.4) |  |
| Regimen |  |  |  | **0.010** |
| Myeloablative conditioning | 454 (49.7) | 258 (57.2) | 712 (52.2) |  |
| Reduced conditioning | 458 (50.2) | 193 (42.8) | 651 (47.7) |  |
| Engraftment (day 0 – 100) |  |  |  | 0.15 |
| Yes | 858 (94.0) | 413 (91.6) | 1 271 (93.2) |  |
| No | 11 (1.2) | 12 (2.7) | 23 (1.7) |  |
| Never below/ missing | 44 (4.8) | 26 (5.8) | 70 (5.1) |  |
| Time to engraftment, median days (IQR) | 15 (12 – 18) | 16 (14 – 19) | 15 (13 – 18) | **<0.001** |
| Acute Graft vs. Host Disease |  |  |  | **<0.001** |
| Grade I/ II | 321 (35.2) | 135 (29.9) | 456 (33.4) |  |
| Grade III/ IV | 73 (8.0) | 82 (18.2) | 155 (11.4) |  |
| Present, grade unknown | 9 (1.0) | 5 (1.1) | 14 (1.0) |  |
| Not evaluated/ missing | 33 (3.6) | 35 (7.8) | 68 (5.0) |  |
| Follow-up time, person-years (IQR) | 2.03  (0.9 – 4.1) | 1.4  (0.5 – 3.2) | 1.8  (0.7 – 4.0) | **0.001** |

Data are n (%) or median (IQR) if indicated, *HCT* haematopoetic cell transplantation, *MDS/ MPN* myelodysplastic syndrome/myeloproliferative neoplasm

°Other: bone marrow failure n=28 (2.1%), inherited disorders n=7 (0.5%), hemoglobinopathies n=1 (0.1%), Infection n=1 (0.1%), other n=2 (0.1%)**.**

**Table S2: Resistance Patterns**

|  | **10/09/2009 –04/02/2015**  **N = 322 (41.2%)** | **05/02/2015 – 09/10/2018**  **N = 459 (58.7%)** | **Total**  **N = 781**  **(100 %)** | **p-value** |
| --- | --- | --- | --- | --- |
| **Coagulase-negative staphylococci** | **N = 107 (33.2)** | **N = 103 (22.4)** | **N = 210 (26.9)** |  |
| Resistant to oxacillin | 105 (98.1) | 100 (97.1) | 205 (97.6) | 0.62 |
| ***Staphylococcus aureus*** | **N = 4 (1.2)** | **N = 5 (1.1)** | **N = 9 (1.2)** |  |
| Resistant to oxacillin | 0 | 0 | 0 |  |
| ***Streptococcus* spp.** | **N = 37 (11.5)** | **N = 40 (8.7)** | **N = 77 (9.9)** |  |
| Resistant to penicillin | 6 (16.2) | 10 (25.0) | 16 (20.7) | 0.41 |
| ***Enterococcus faecium*** | **N = 54 (16.8)** | **N = 62 (13.5)** | **N = 116 (14.9)** |  |
| Resistant to vancomycin | 0 | 1 (1.6) | 1 (0.9) | 0.35 |
| Resistant to daptomycin | 1 (1.9) | 2 (3.3) | 3 (2.6) | 0.97 |
| ***Enterococcus faecalis*** | **N = 9 (2.8)** | **N = 8 (1.7)** | **N = 17 (2.2)** |  |
| Resistant to ampicillin | 2 (22.0) | 0 | 2 (11.0) | 0.13 |
| ***Enterobacterales*** | **N = 59 (18.3)** | **N = 136 (29.6)** | **N = 195 (25.0)** |  |
| Resistant to ciprofloxacin | 25 (42.4) | 56 (41.2) | 81 (41.5) | 0.85 |
| Resistant to cefepime | 11 (18.6) | 22 (16.2) | 33 (16.9) | 0.68 |
| Resistant to piperacillin/ tazobactam | 10 (16.9) | 22 (16.2) | 32 (16.4) | 0.93 |
| Resistant to meropenem | 2 (3.4) | 0 | 2 (1.0) |  |
| ESBL-producing | 13 (22.0) | 28 (20.6) | 41 (21.0) | 0.72 |
| Chromosomal AmpC | 5 (8.5) | 8 (5.9) | 13 (6.7) | 0.52 |
| Carbapenemases | 0 | 0 | 0 |  |
| ***Pseudomonas aeruginosa*** | **N = 18 (5.6)** | **N = 31 (6.8)** | **N = 49 (6.3)** |  |
| Resistant to ciprofloxacin | 4 (22.2) | 7 (22.6) | 11 (22.4) | 0.91 |
| Resistant to piperacillin/ tazobactam | 2 (11.1) | 10 (32.2) | 12 (24.5) | 0.12 |
| Resistant to cefepime | 2 (11.1) | 4 (12.9) | 6 (12.2) | 0.91 |
| Resistant to meropenem | 4 (22.2) | 6 (19.3) | 10 (20.4) | 0.82 |
| Resistant to piperacillin/ tazobactam, cefepime and carbapenems | 2 (11.1) | 2 (6.5) | 4 (8.2) | 0.85 |
| **Anaerobes** | **N = 5 (1.6)** | **N = 21 (4.6)** | **N = 26 (3.3)** |  |
| Resistant to penicillin | 0 | 12 (57.0) | 12 (46.0) | **0.05** |
| ***Candida* *albicans*** | **N = 2 (0.6)** | **N = 1 (0.2)** | **N = 3 (0.4)** |  |
| Resistant to fluconazol | 0 | 0 | 0 |  |
| ***Candida* non-*albicans*** | **N = 11 (3.4)** | **N = 9 (2.0)** | **N = 20 (2.6)** |  |
| Resistant to fluconazol | 8 (72.7) | 7 (77.7) | 15 (75.0) | 0.44 |
| Resistant to caspofungin | 1 (9.0) | 1 (11.1) | 2 (10.0) | 0.70 |
| **Other pathogens** | **N = 16 (5.0)** | **N = 43 (9.4)** | **N = 59 (7.6)** |  |

**Table S3: Uni- and multivariate cause specific cox regression analysis for the endpoint of first bloodstream infection in the presence of the competing risk of death**

|  | **Univariate analysis** | | | **Multivariate analysis** | | | |
| --- | --- | --- | --- | --- | --- | --- | --- |
|  | **HR (95% CI)** | **p- value** | | **HR (95% CI)** | | **p- value** | |
| Gender, male vs. female | 1.28 (1.03 – 1.59) | 0.025 | 1.26 (1.01 – 1.57) | | **0.039** | |  |
| Age, ≥60 vs. <60 years | 1.08 (0.86 – 1.35) | 0.524 | 1.04 (0.81 – 1.34) | | 0.749 | |  |
| Karnofsky score at transplantation°, ≥90% vs. <90% | 0.73 (0.57 – 0.95) | 0.017 | 0.79 (0.61 – 1.03) | | 0.083 | |  |
| Comorbidities, present vs. absent | 1.52 (1.24 – 1.87) | <0.001 | 1.48 (1.19 – 1.83) | | **<0.001** | |  |
| AML, present vs. other haematological conditions | 1.05 (0.86 – 1.29) | 0.636 | 1.05 (0.85 – 1.30) | | 0.658 | |  |
| Conditioning regimen  MAC vs. RIC |  |  |  | |  | |  |
| -14d – 30d | 1.69 (1.30 – 2.21) | <0.001 | 1.82 (1.38 – 2.42) | | **<0.001** | |  |
| 31d – 365d | 0.74 (0.52 – 1.06) | 0.103 | 0.91 (0.62 – 1.33) | | 0.626 | |  |
| Graft cell source, cord blood vs. other graft sources | 2.61 (0.98 – 7.01) | 0.056 | 2.18 (0.80 – 5.95) | | 0.128 | |  |
| HLA- match, unrelated vs. related donor | 1.2 (0.97 – 1.48) | 0.088 | 1.13 (0.91 – 1.40) | | 0.276 | |  |
| Neutrophil recovery°  post-engraftment vs. aplasia | 0.39 (0.24 – 0.64) | <0.001 | 0.44 (0.26 – 0.73) | | **0.002** | |  |
| aGvHD°, present vs. absent |  |  |  | |  | |  |
| Grade I/II | 1.44 (0.94 – 2.20) | 0.091 | 1.48 (0.96 – 2.27) | | 0.078 | |  |
| Grade III/IV | 6.55 (4.50 – 9.53) | <0.001 | 6.62 (4.52 – 9.69) | | **<0.001** | |  |
| Progression/relapse of haematological disease°, present vs. absent | 2.84 (1.87 – 4.29) | <0.001 | 3.11 (2.04 – 4.73) | | **<0.001** | |  |

*AML* acute myeloid leukemia; *aGVHD* acute graft-versus-host-disease; *HCT* haematopoietic cell transplantation; *MAC* myeloablative conditioning; *RIC* reduced-intensity conditioning, °Time- dependent covariates. Non-time- dependent covariates are assessed at the time point of first HCT

**Table S4: Uni- and multivariate cox regression analysis for the endpoint of mortality**

|  | **Univariate analysis** | | **Multivariate analysis** | | |
| --- | --- | --- | --- | --- | --- |
|  | **HR (95% CI)** | **p- value** | **HR (95% CI)** | **p- value** | |
| Gender, male vs. female | 1.02 (0.82 – 1.27) | 0.857 | 0.97 (0.78 – 1.21) | 0.775 |  |
| Age, ≥60 vs. <60 years | 1.31 (1.05 – 1.64) | 0.017 | 1.07 (0.84 – 1.37) | 0.561 |  |
| Karnofsky score at transplantation°, ≥90% vs. <90% | 0.47 (0.37 – 0.59) | <0.001 | 0.55 (0.43 – 0.71) | **<0.001** |  |
| Comorbidities, present vs. absent | 1.63 (1.32 – 2.01) | <0.001 | 1.34 (1.07 – 1.67) | **0.011** |  |
| AML, present vs. other haematological conditions | 1.03 (0.83 – 1.27) | 0.789 | 0.86 (0.69 – 1.07) | 0.167 |  |
| Number of HCT°, >1 vs. 1 | 3.8 (1.95 – 7.39) | <0.001 | 1.37 (0.67 – 2.79) | 0.389 |  |
| Conditioning regimen, MAC vs. RIC | 0.72 (0.59 – 0.89) | 0.003 | 0.89 (0.70 – 1.12) | 0.311 |  |
| Graft cell source, cord blood vs. other graft sources | 1.19 (0.30 – 4.78) | 0.805 | 0.81 (0.20 – 3.27) | 0. 762 |  |
| aGvHD°, present vs. absent |  |  |  |  |  |
| Grade I/II | 0.9 (0.68 – 1.20) | 0.485 | 0.91 (0.68 – 1.21) | 0.517 |  |
| Grade III/IV | 2.84 (2.14 – 3.78) | <0.001 | 2.39 (1.77 – 3.24) | **<0.001** |  |
| Progression/Relapse of haematological disease°, present vs. absent | 10.0 (7.94 – 12.6) | <0.001 | 9.4 (7.41 – 11.91) | **<0.001** |  |
| HLA- match, unrelated vs. related donor |  |  |  |  |  |
| -14 – 30 days | 3.17 (1.05 – 9.57) | 0.040 | 2.9 (0.96 – 8.75) | 0.059 |  |
| 31 – 100 days | 1.49 (0.97 – 2.31) | 0.072 | 1.61 (1.03 – 2.51) | **0.037** |  |
| 101 – 365 days | 1.18 (0.92 – 1.53) | 0.197 | 1.16 (0.89 – 1.50) | 0.270 |  |
| Bloodstream infection °, present vs. absent |  |  |  |  |  |
| -14 – 30 days | 6.06 (2.42 – 15.17) | <0.001 | 5.95 (2.38 – 14.86) | **<0.001** |  |
| 31 – 100 days | 7.34 (3.80 – 14.18) | <0.001 | 4.41 (2.33 – 8.34) | **<0.001** |  |
| 101 – 365 days | 20.57 (15.2 – 27.8) | <0.001 | 10.7 (7.8 – 14.7) | **<0.001** |  |

*AML* acute myeloid leukemia; *aGVHD* acute graft-versus-host-disease; *HCT* haematopoietic cell transplantation; *MAC* myeloablative conditioning; *RIC* reduced-intensity conditioning

°Time-dependent covariates

Non-time- dependent covariates are assessed at the timepoint of first HCT

**Figure S1: Consort diagram of HCT and patients**


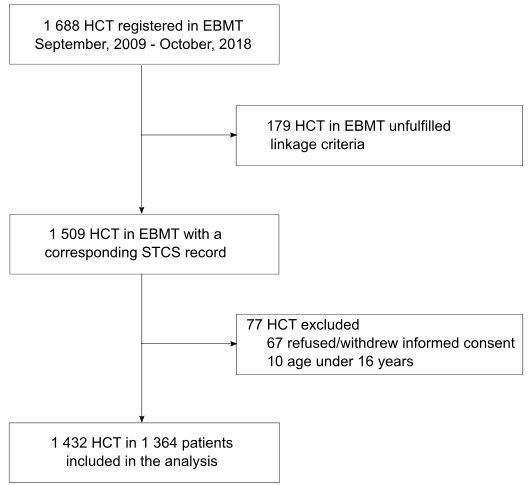


HCT haematopoetic cell transplantation; STCS Swiss Transplant Cohort Study; EBMT European Society for Blood and Marrow Transplantation

**Figure S2: Cumulative incidence of bloodstream infections**


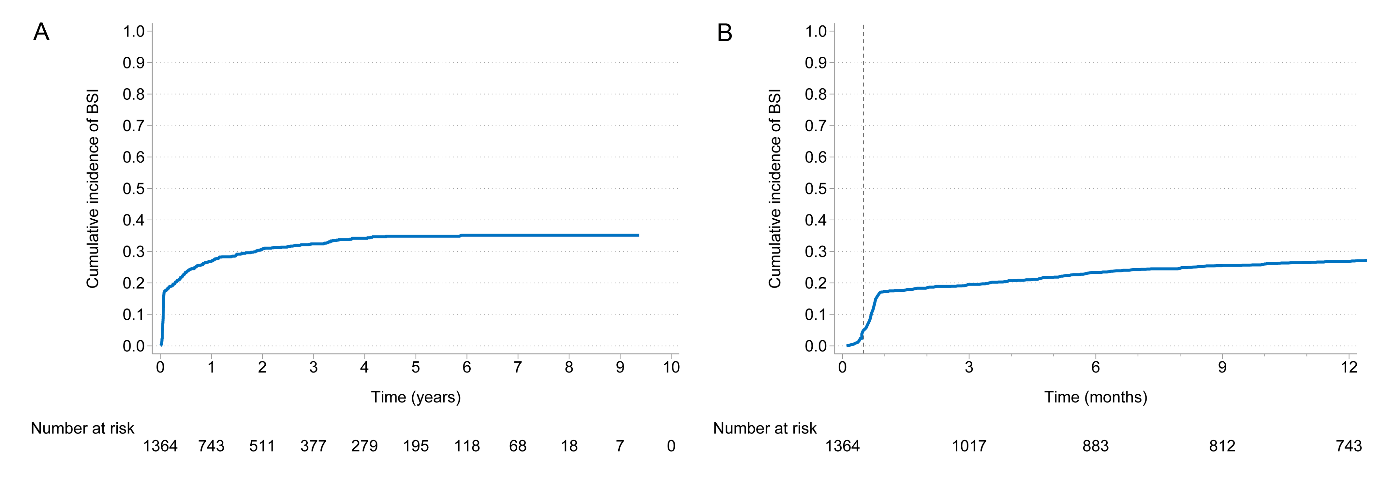


A. Cumulative incidence of BSI over the entire follow-up time, accounting for competing risk of death

B. Cumulative incidence of BSI over the entire follow-up time, accounting for competing risk of death. Dotted line represents the transplantation day.

**Figure S3: Overall survival after HCT during the entire follow-up period in the STCS**


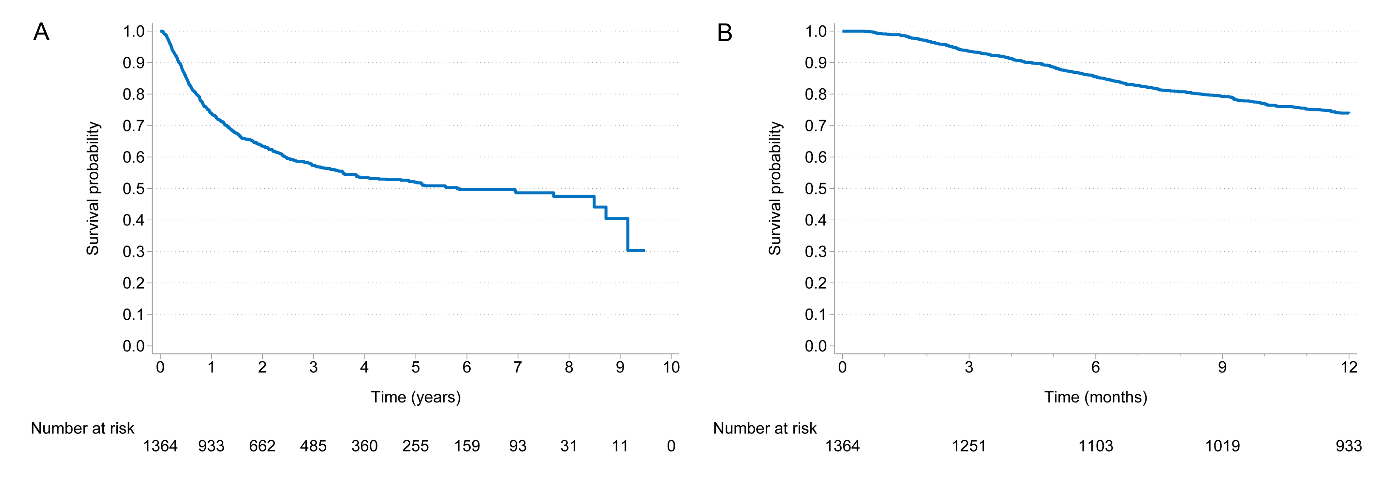


Kaplan-Meier-plot showing overall survival. HCT haematopoetic cell transplantation; STCS Swiss Transplant Cohort Study
